# Hydrogen Sulfide Inhibits High-Salt Diet-Induced Myocardial Oxidative Stress and Myocardial Hypertrophy in Dahl Rats

**DOI:** 10.3389/fphar.2017.00128

**Published:** 2017-03-16

**Authors:** Pan Huang, Zhizhou Shen, Wen Yu, Yaqian Huang, Chaoshu Tang, Junbao Du, Hongfang Jin

**Affiliations:** ^1^Department of Pediatrics, Peking University First HospitalBeijing, China; ^2^Department of Physiology and Pathophysiology, Peking University Health Science CentreBeijing, China; ^3^Key Laboratory of Molecular Cardiology, Ministry of Education, Peking UniversityBeijing, China

**Keywords:** hydrogen sulfide, high salt diet, salt sensitive hypertension, myocardial hypertrophy, oxidative stress

## Abstract

The study aimed to examine the protective effect of hydrogen sulfide (H_2_S) on high-salt-induced oxidative stress and myocardial hypertrophy in salt-sensitive (Dahl) rats. Thirty male Dahl rats and 40 SD rats were included in the study. They were randomly divided into Dahl control (Dahl + NS), Dahl high salt (Dahl + HS), Dahl + HS + NaHS, SD + NS, SD + HS, SD + HS + NaHS, and SD + HS + hydroxylamine (HA). Rats in Dahl + NS and SD + NS groups were given chow with 0.5% NaCl and 0.9% normal saline intraperitoneally daily. Myocardial structure, α-myosin heavy chain (α-MHC) and β-myosin heavy chain (β-MHC) expressions were determined. Endogenous myocardial H_2_S pathway and oxidative stress in myocardial tissues were tested. Myocardial H_2_S pathway was downregulated with myocardial hypertrophy featured by increased heart weight/body weight and cardiomyocytes cross-sectional area, decreased α-MHC and increased β-MHC expressions in Dahl rats with high-salt diet (all *P* < 0.01), and oxidative stress in myocardial tissues was significantly activated, demonstrated by the increased contents of hydroxyl radical, malondialdehyde and oxidized glutathione and decreased total antioxidant capacity, carbon monoxide, catalase, glutathione, glutathione peroxidase, superoxide dismutase (SOD) activities and decreased SOD1 and SOD2 protein expressions (*P* < 0.05, *P* < 0.01). However, H_2_S reduced myocardial hypertrophy with decreased heart weight/body weight and cardiomyocytes cross-sectional area, increased α-MHC, decreased β-MHC expressions and inhibited oxidative stress in myocardial tissues of Dahl rats with high-salt diet. However, no significant difference was found in H_2_S pathway, myocardial structure, α-MHC and β-MHC protein and oxidative status in myocardial tissues among SD + NS, SD + HS, and SD + HS + NaHS groups. HA, an inhibitor of cystathionine β-synthase, inhibited myocardial H_2_S pathway (*P* < 0.01), and stimulated myocardial hypertrophy and oxidative stress in SD rats with high-salt diet. Hence, H_2_S inhibited myocardial hypertrophy in high salt-stimulated Dahl rats in association with the enhancement of antioxidant capacity, thereby inhibiting oxidative stress in myocardial tissues.

## Introduction

Myocardial hypertrophy is a common cardiovascular pathology, which is an independent risk factor of cardiovascular disease (Cramariuc and Gerdts, 2016). Clinical and basic studies have shown that long-term high-salt intake is an independent risk factor of myocardial hypertrophy (Mimran and du Cailar, 2008; Li et al., 2009). The study showed that salt-sensitive hypertensive patients were more prone to develop myocardial hypertrophy and cardiovascular events (Chamarthi et al., 2010). Previous studies suggested that the potential mechanisms underlying high-salt-induced myocardial hypertrophy mainly included hemodynamic changes, sodium serum levels, increased sodium influx of cardiomyocytes, activation of cardiac renin-angiotensin-aldosterone system (RAAS) and activation of cardiac sympathetic nervous system (Gallo et al., 1990; Galderisi et al., 1991; Harmsen and Leenen, 1992; Le Corvoisier et al., 2010; He and Macgregor, 2012). However, the mechanism for high-salt-induced myocardial hypertrophy still remains unclear.

Endogenous hydrogen sulfide (H_2_S) can be produced with L-cysteine as the substrate, catalyzed by the key enzymes cystathionine β-synthase (CBS) and cystathionine γ-lyase (CSE), and it can also be produced with 3-mercaptopyruvate as the substrate, catalyzed by mercaptopyruvate sulfurtransferase (MPST). The three enzymes are richly expressed in the cardiovascular system (Geng et al., 2004b). Studies showed that H_2_S had a protective effect on myocardial injury in different diseases (Liu et al., 2014; Shen et al., 2015). H_2_S protected the myocardium and cardiac function by inhibiting inflammatory reaction and apoptosis, etc (Sodha et al., 2009; Su et al., 2009; Wei et al., 2010; Lu et al., 2013; Yang et al., 2014). Furthermore, our previous studies demonstrated that down-regulation of renal H_2_S/CBS pathway induced by high salt led to the development of hypertension, and H_2_S could improve aortic structural remodeling and reduce the high salt-induced renal injury in Dahl rats (Huang et al., 2015, 2016), suggesting that the effect of H_2_S on the function and structure of aorta and kidney participated in the protective regulation of H_2_S on the myocardial hypertrophy. However, it is unclear whether endogenous H_2_S pathway was involved in the pathogenesis of high salt-induced myocardial hypertrophy.

Considering that the oxidative stress injury is a key pathological basis of myocardial hypertrophy, we focused on the myocardial oxidative stress as the target in salt-sensitive animal model and designed the present study to explore the role of H_2_S in myocardial hypertrophy induced by high-salt and the possible mechanism underlying myocardial hypertrophy in rats.

## Materials and Methods

### Animal Protocol

Thirty male Dahl rats and 40 SD rats (Charles River Laboratory Animal Technology Co., Ltd., China; license number: SCXK 2012-0001) at 6 weeks of age were randomly divided into the following groups including Dahl control group (Dahl + NS), Dahl high salt group (Dahl + HS), Dahl high salt + NaHS group (Dahl + HS + NaHS), SD control group (SD + NS), SD high salt group (SD + HS), SD high salt + sodium hydrosulfide (NaHS) group (SD + HS + NaHS) and SD high salt + hydroxylamine (HA) group (SD + HS + HA). Rats in Dahl + NS and SD + NS groups were given chow with 0.5% sodium chloride (NaCl) and 0.9% saline by intraperitoneal injection daily. Rats in Dahl + HS and SD + HS groups were given chow with 8% NaCl and 0.9% saline by intraperitoneal injection daily. Rats in the Dahl + HS + NaHS and SD + HS + NaHS groups were given chow with 8% NaCl and 90 μmol/kg NaHS by intraperitoneal injection daily (Shi et al., 2007). Rats in the SD + HS + HA group were given chow with 8% NaCl and 12.5 mg/kg HA (an inhibitor of CBS) per day (Han et al., 2005) and NaHS and HA solutions were freshly prepared with 0.9% saline daily. All rats were housed in Animal Center of Peking University First Hospital, freely drinking water and food throughout the whole experiment. The rats were kept at 25°C constant temperature and circadian rhythm of 12 h/12 h. The animal experiment was approved by the Experimental Animal Ethics Committee of Peking University First Hospital and strictly followed the regulations and guidelines of experimental animals of Peking University First Hospital.

### Measurement of Body Weight and Heart Weight

At the end of the experiment, the body weight of conscious and quiet rats was measured. The rats were anesthetized by intraperitoneal injection of 25% urethane at 1.25 g/kg body weight. After the chest was opened, the hearts were removed rapidly and the heart weight was measured.

### Myocardial Pathology

Appropriately 2 mm of cardiac cross-section tissue was taken, fixed in 4% paraformaldehyde and paraffin-embedded. Then, 5 μm of slice was taken for hematoxylin-eosin (HE) staining. Image acquisition and quantitative analysis of cardiomyocyte HE staining were performed by Leica Q550CW Image Processing and Analysis System. Each sample was randomly selected three fields from the left ventricular tissue, which were used to calculate the size of myocardial cells. The tissue samples of paraffin sections were put in xylene for 5 min, totally three times, then 100, 95, and 75% ethanol for 5 min separately, deionized water for 5 min, totally three times and then stained with Dio solution of 25 μmol/L overnight at 4°C. Fluorescence image acquisition and analysis were performed by using a laser scanning confocal microscope (Olympus, Japan). Three fields were randomly taken from each sample, and approximately 100 cells were quantitatively analyzed for cell size (Wu et al., 2012).

### Real-Time Quantitative Polymerase Chain Reaction (RT-PCR)

Total ribonucleic acid (RNA) was extracted from myocardial tissues by Trizol method. The purity and concentration of the total RNA were determined by the quantitative detection of nucleic acid protein. Two microgram of total RNA was used for complementary deoxyribonucleic acid (cDNA) synthesis by reverse transcription. cDNA synthesis conditions included 25 μL of reaction system for denaturation at 70°C for 5 min, and then at 42°C reaction for 60 min in PCR instrument. Twenty-five microliter of mixture was expanded in an ABI Prism 7300 instrument (Applied Biosystems, Foster, CA, USA). RT-PCR reaction conditions were set at 95°C pre-denaturation for 10 min, 95°C denaturation for 15 s, 60°C annealing for 1 min, and 72°C polymerization extension for 30 s, with a total of 40 cycles. β-actin was used as an internal reference for the calibration. The primers were synthesized by Sangon Biotech (Shanghai) Co., Ltd. The primer sequences were shown in **Table 1**. The ABI prism 7300 system software was for Ct and 2^-ΔΔt^ data analysis (Luo et al., 2013).

**Table 1:** Sequence of targeted gene and β-actin cDNA primer and Taqman

Target	primer	Sequence (5′-3′)	Product
Rat CBS	Forward	CTCCGGGAGAAGGGTTTTGA	81 bp
	Reverse	CATGTTCCCGAGAGTCACCAT
	TaqMan	AGGCACCTGTGGTCAACGAGTCTGG
	probe
Rat CSE	Forward	GCTGAGAGCCTGGGAGGATA	92 bp
	Reverse	TCACTGATCCCGAGGGTAGCT
	TaqMan	CTGAGCTTCCAGCAATCATGACCCATG
	probe
Rat MPST	Forward	CGGCGCTTCCAGGTAGTG	131 bp
	Reverse	CTGGTCAGGAATTCAGTGAATGG
	TaqMan	CCGCGCAGCTGGCCGTTT
	probe
Rat β-actin	Forward	ACCCGCGAGTACAACCTTCTT	80 bp
	Reverse	TATCGTCATCCATGGCGAACT
	TaqMan	CCTCCGTCGCCGGTCCACAC
	probe

### Western Blot Assay

Myocardial tissues were ground into homogenate according to the mass volume ratio 1:10 (mg/μL) adding 1 × tissue lysate, and then the homogenate was centrifuged for 10 min at 4°C for supernatant. The supernatant was collected and added into the same volume of 2 × loading buffer at 100°C for 5 min. Each channel was added with equal amount of protein mixture for polyacrylamide gel electrophoresis, transferred into membrane. The primary antibodies including α-myosin heavy chain (α-MHC; 1:200, Santa Cruz Biotechnology, USA), β-MHC (1:200, Santa Cruz Biotechnology, USA), CBS (1:2000, Santa Cruz Biotechnology, USA), CSE (1:500, Sigma, USA), MPST (1:4000, Santa Cruz Biotechnology, USA), superoxide dismutase 1 (SOD1; 1:5000, Stressgen, USA), SOD2 (1:5000, Stressgen, USA), β-actin (1:2000, Santa Cruz Biotechnology, USA), and glyceraldehyde-3-phosphate dehydrogenase (GAPDH; 1:2000, Kangcheng, Shanghai, China) were added into membrane overnight at 4°C for incubation, and then washed by TTBS for 10 min/time of total of four times, with secondary antibodies for 1 h at room temperature for full incubation. AlphaImager gel imaging system was used to scan the protein band and measure the optical density of the protein band, and β-actin and GAPDH as internal reference protein were used to correct (Luo et al., 2013).

### H_2_S Content in Myocardial Tissues Assay

Appropriate amount of myocardial tissues was weighed and placed in 1.5 ml of EP tube, according to the mass volume ratio (1/10) with phosphate buffer saline (PBS) solution (pH7.2, 50 mmol/L), then myocardial tissues were ground into homogenate which was centrifuged at 12000 g for 10 min at 4°C for the supernatant, and still then the supernatant was measured to protein content. The H_2_S content in the supernatant was determined using a Free Radical Detection Analyzer TBR4100 (World Precision Instruments, Shanghai, China) (Fox et al., 2012). At First, a 100 μm of ISO-H_2_S-100 sensor (ISO-H_2_S-100, WPI, Shanghai, China) was polarized in PBS buffer solution (pH 7.2, 50 mmol/L). The sensor could be calibrated as a stable reference current came out (usually 100–2000 pA). Approximately 10 mm of the sensor tip was immersed into 20 ml of PBS buffer solution (pH 7.2, 50 mmol/L) until a steady current appeared on the display. Six different concentrations of sodium sulfide solution (1, 2, 4, 8, 16, and 32 μmol/L) prepared in PBS buffer were successively detected by the detecting probe. After each sample added, the current output rapidly increased to the plateau. As long as the current reached the plateau, the next sample could be detected. The calibration curve was prepared from the signal output (pA) and the corresponding H_2_S concentration (μM). The length of immersion was about 10 mm for each sample. The H_2_S content in each sample could be calculated according to the calibration curve of pA-H_2_S concentration.

### Oxidative Stress Indices in Myocardial Tissues Assay

Appropriate amount of myocardial tissues was put into PBS buffer (pH 7.2, 10 mmol/L) according to the mass ratio of 1:10 (mg/μl), and was fully ground into homogenate which was centrifuged at 12000 *g* for 10 min at 4°C for the supernatant. The activities of SOD, catalase (CAT), glutathione peroxidase (GSH-PX), and oxidized glutathione (GSSG) were determined by biochemical colorimetry. Glutathione (GSH), malondialdehyde (MDA), hydroxyl radical (⋅OH), carbon monoxide (CO), and total antioxidant capacity (T-AOC) were measured by biochemical colorimetry. The experimental procedure was strictly carried out according to the kit (Nanjing Jiancheng Biology Engineering Research Institute, China).

### Statistical Analysis

The data statistical analysis was performed using ABI SPSS 13.0 software. The results was expressed as mean ± standard error (mean ± SEM). The means among more than 2 groups were compared using one-way ANOVA. *P* < 0.05 was significant.

## Results

### Effect of High-Salt Diet on H_2_S/CBS Pathway of Myocardial Tissues in Dahl Rats

**FIGURE 1:**
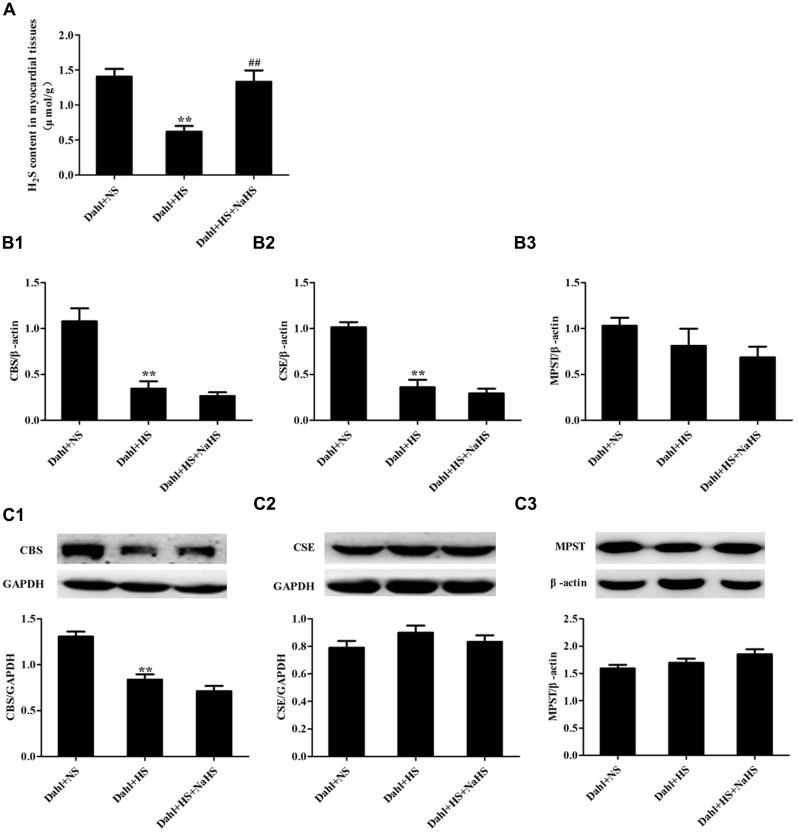
**The change in H_2_S pathway of myocardial tissues in each group of Dahl rats. (A)** H_2_S content of myocardial tissues in Dahl rats. **(B1–B3)** CBS, MPST, and CSE mRNA expressions of myocardial tissues in Dahl rats. **(C1–C3)** CBS, MPST, and CSE protein expressions of myocardial tissues in Dahl rats. The data are described as mean ± SEM, *n* = 10, ^∗∗^*P* < 0.01 versus Dahl + NS, ^##^*P* < 0.01 versus Dahl + HS.

**FIGURE 2:**
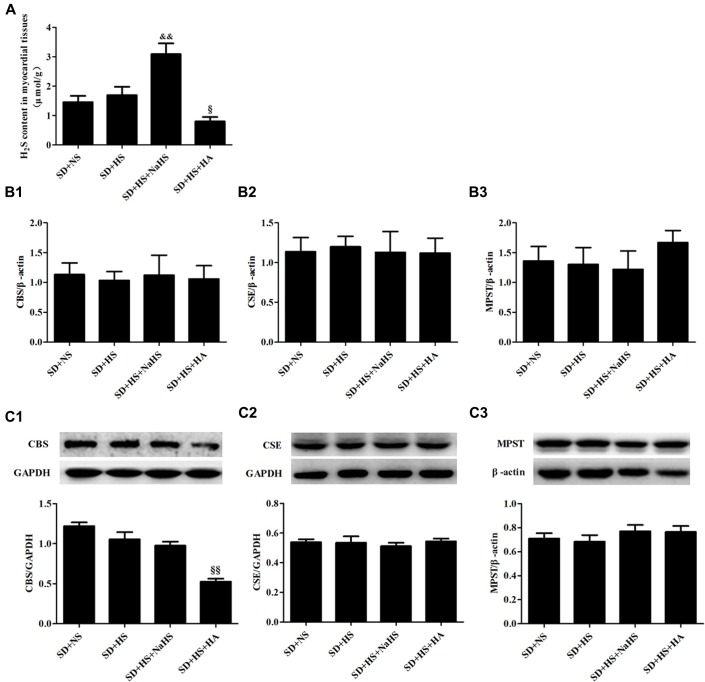
**The change in H_2_S pathway of myocardial tissues in each group of SD rats. (A)** H_2_S content of myocardial tissues in SD rats. **(B1–B3)** CBS, MPST, and CSE mRNA expressions of myocardial tissues in SD rats. **(C1–C3)** CBS, MPST, and CSE protein expressions of myocardial tissues in SD rat. The data are described as mean ± SEM, *n* = 10, ^&&^*P* < 0.01, ^§^ *P* < 0.05, ^§§^ *P* < 0.01 versus SD + HS.

Compared with rats in Dahl + NS group, H_2_S content of myocardial tissues was significantly decreased (*P* < 0.01; **Figure 1A**) with downregulated CBS mRNA and protein expression (*P* < 0.01; **Figures 1B1,C1**) and reduced CSE mRNA (*P* < 0.01; **Figure 1B2**), but CSE protein expression and MPST mRNA and protein expressions of myocardial tissues did not differ in Dahl + HS group rats (*P* > 0.05; **Figures 1B3,C2,C3**). There was no obvious difference in H_2_S content and CBS, CSE and MPST mRNA and protein expressions of myocardial tissues among SD + NS group, SD + HS group and SD + HS + NaHS group rats (*P* > 0.05; **Figures 2A–C**). But, compared with SD rats, H_2_S content and CBS protein expression of myocardial tissues were significantly reduced (*P* < 0.01; **Figures 2A,C1**), whereas CBS mRNA, CSE and MPST mRNA and protein expressions did not change in SD rats treated with HA (*P* > 0.05; **Figures 2B,C2,C3**).

### Effect of H_2_S on High-Salt-Induced Myocardial Hypertrophy in Dahl + HS Group Rats

**FIGURE 3:**
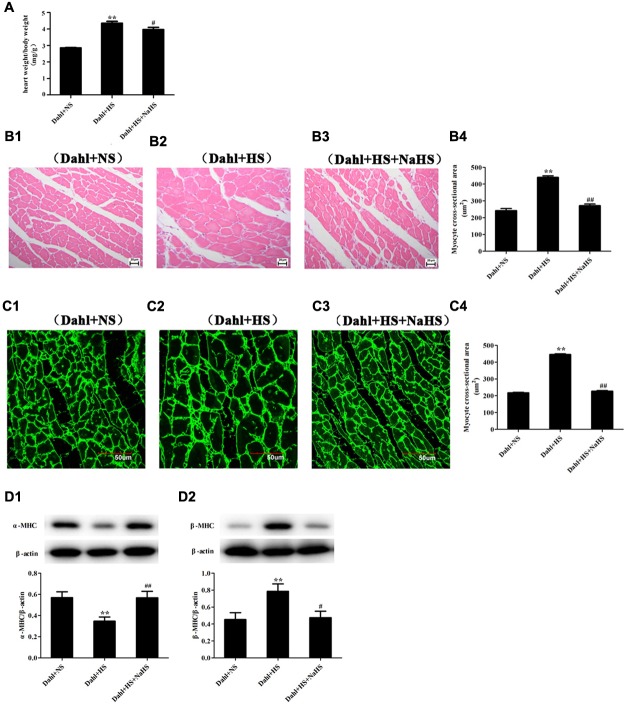
**Myocardial structure change in each group of Dahl rats. (A)** heart weight/body weight in Dahl rat. **(B1–B4)** cardiomyocyte HE staining (400×) in Dahl rats. **(C1–C4)** cardiomyocyte Dio staining (600×) in Dahl rats. **(D1–D2)** α-MHC and β-MHC protein expressions of myocardial tissues in Dahl rats. The data are described as mean ± SEM, *n* = 10, ^∗∗^*P* < 0.01 versus Dahl + NS, ^#^*P* < 0.05, ^##^*P* < 0.01 versus Dahl + HS.

Compared with rats in Dahl + NS group, those in Dahl + HS group showed marked myocardial hypertrophy including increased heart weight/body weight (*P* < 0.01; **Figure 3A**) and cardiomyocyte cross-sectional area (*P* < 0.01; **Figures 3B1–B4,C1–C4**), decreased α-MHC protein, but increased β-MHC protein (*P* < 0.01; **Figures 3D1,D2**). However, compared with rats in Dahl + HS group, those in Dahl + HS + NaHS group exhibited lessened myocardial hypertrophy characterized by decreased heart weight/body weight (*P* < 0.05; **Figure 3A**), reduced cardiomyocyte cross-sectional area (*P* < 0.01; **Figures 3B1–B4,C1–C4**), increased α-MHC protein, but decreased β-MHC protein (*P* < 0.05, *P* < 0.01; **Figures 3D1,D2**).

### HA Induced Myocardial Hypertrophy in SD + HS Group Rats

**FIGURE 4:**
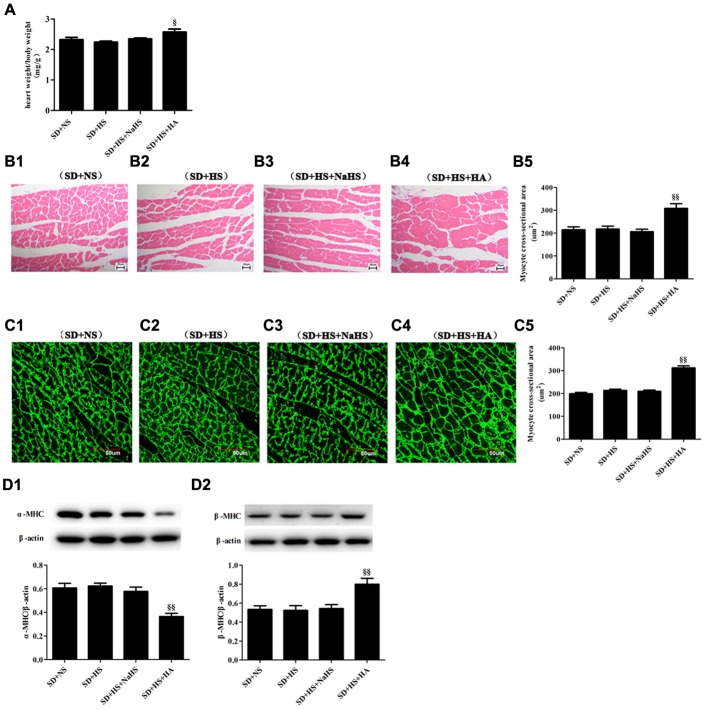
**Myocardial structure change in each group of SD rats. (A)** heart weight/body weight in SD rats. **(B1–B5)** cardiomyocyte HE staining (400×) in SD rats. **(C1–C5)** cardiomyocyte Dio staining (600×) in SD rats. **(D1–D2)** α-MHC and β-MHC protein expressions of myocardial tissues in SD rats. The data are described as mean ± SEM, *n* = 10, ^§^ *P* < 0.05, ^§§^ *P* < 0.01 versus SD + HS.

There was no statistical difference in myocardial structure, and α-MHC and β-MHC protein expressions of myocardial tissues of rats among SD + NS group, SD + HS group and SD + HS + NaHS group (*P* > 0.05; **Figures 4A–D**). However, compared with those in rats of SD + HS group, ratio of heart weight/body weight and cardiomyocyte cross-sectional area were increased, α-MHC protein was downregulated but β-MHC protein expression was upregulated in myocardial tissues of rats in SD + HS + HA group (*P* < 0.05, *P* < 0.01; **Figures 4A–D**).

### Effect of H_2_S on High-Salt-Induced Excessive Activation of Oxidative Stress of Myocardial Tissues in Dahl Rats

**FIGURE 5:**
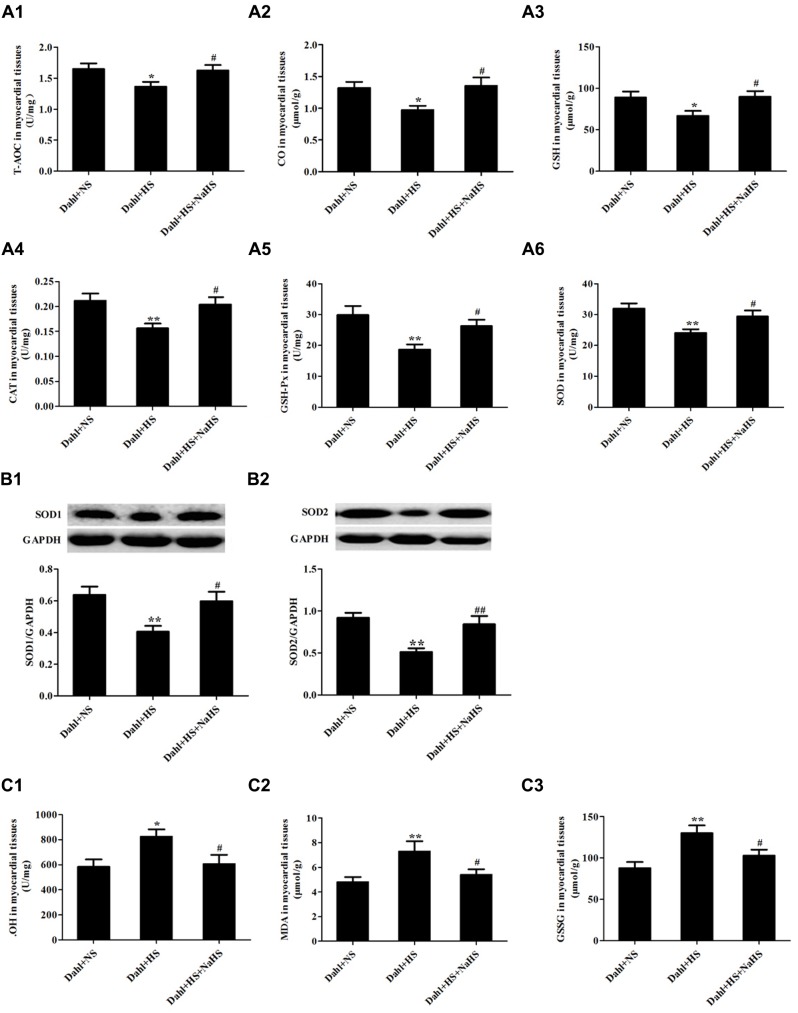
**Oxidative stress and antioxidant stress indices of myocardial tissues in each group of Dahl rats. (A1)** T-AOC of myocardial tissues in Dahl rats. **(A2)** CO of myocardial tissues in Dahl rats. **(A3)** GSH myocardial tissues in Dahl rats. **(A4)** CAT activity of myocardial tissues in Dahl rats. **(A5)** GSH-Px activity of myocardial tissues in Dahl rats. **(A6)** SOD activity of myocardial tissues in Dahl rats. **(B1,B2)** SOD1 and SOD2 protein expressions of myocardial tissues in Dahl rats. **(C1)** ⋅OH content of myocardial tissues in Dahl rats. **(C2)** MDA content of myocardial tissues in Dahl rats. **(C3)** GSSG content of myocardial tissues in Dahl rats. The data are described as mean ± SEM, *n* = 10, ^∗^*P* < 0.05, ^∗∗^*P* < 0.01 versus Dahl + NS, ^#^*P* < 0.05, ^##^*P* < 0.01, versus Dahl + HS.

Compared with rats of Dahl + NS group, the levels of T-AOC, CO and GSH, the activities of CAT, GSH-Px and SOD (*P* < 0.05, *P* < 0.01; **Figures 5A1–A6**) and protein expressions of SOD1 and SOD2 were decreased (*P* < 0.01; **Figures 5B1,B2**), but the contents of ⋅OH, MDA, and GSSG were obviously increased (*P* < 0.05, *P* < 0.01; **Figures 5C1–C3**) in the myocardial tissues of rats in Dahl + HS group. Compared with rats of Dahl + HS group, the levels of T-AOC, CO, and GSH, the activities of CAT, GSH-Px, and SOD (*P* < 0.05; **Figures 5A1–A6**), and protein expressions of SOD1 and SOD2 were significantly increased (*P* < 0.05, *P* < 0.01; **Figures 5B1,B2**), but the contents of ⋅OH, MDA, and GSSG were significantly decreased (*P* < 0.05; **Figures 5C1–C3**) in myocardial tissues of rats in Dahl + HS + NaHS group.

### HA Activated Oxidative Stress of Myocardial Tissues in SD + HS Group Rats

**FIGURE 6:**
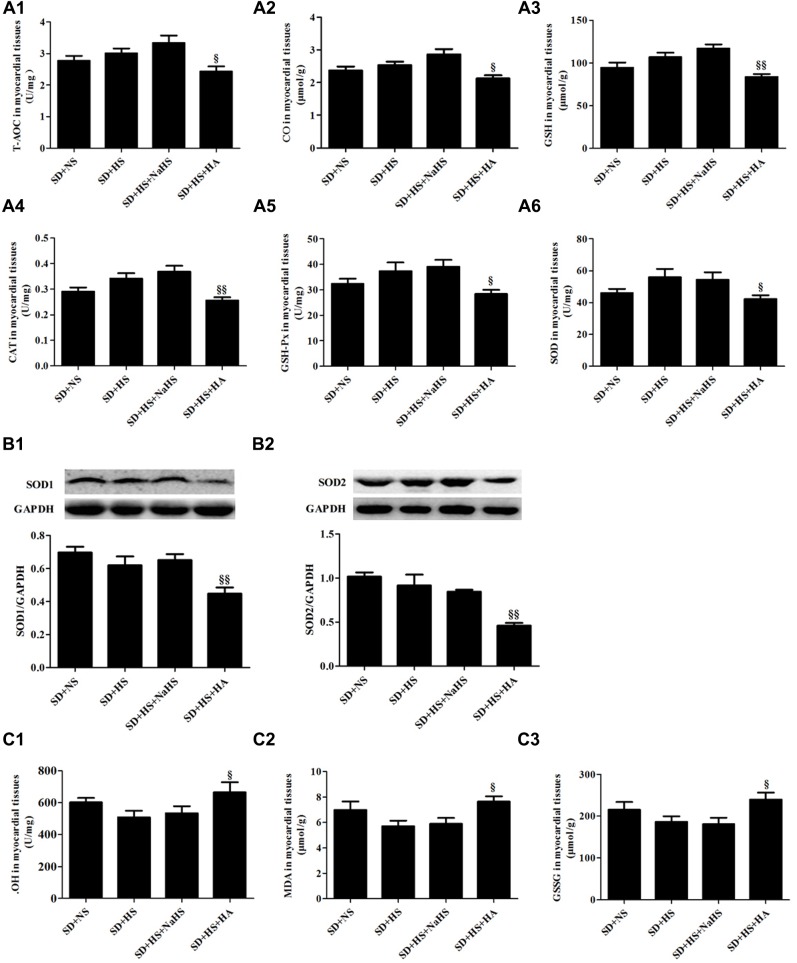
**Oxidative stress and antioxidant stress indices of myocardial tissues in each group of SD rats. (A1)** T-AOC of myocardial tissues in SD rats. **(A2)** CO of myocardial tissues in SD rats. **(A3)** GSH of myocardial tissues in SD rats. **(A4)** CAT activity of myocardial tissues in SD rats. **(A5)** GSH-Px activity of myocardial tissues in SD rats. **(A6)** SOD activity of myocardial tissues in SD rats. **(B1,B2)** SOD1 and SOD2 protein expressions of myocardial tissues in SD rats. **(C1)** ⋅OH content of myocardial tissues in SD rats. **(C2)** MDA content of myocardial tissues in SD rats. **(C3)** GSSG content of myocardial tissues in SD rats. The data are described as mean ± SEM, *n* = 10, ^§^ *P* < 0.05, ^§§^ *P* < 0.01 versus SD + HS.

There was no significant difference in the levels of T-AOC, CO and GSH, the activities of CAT, GSH-Px and SOD, protein expressions of SOD1 and SOD2 (*P* > 0.05; **Figures 6A1–A6,B1,B2**) as well as the contents of ⋅OH, MDA, and GSSG of myocardial tissues (*P* > 0.05; **Figures 6C1–C3**) among SD + NS group, SD + HS group and SD + HS + NaHS group of rats. However, compared with those of SD + HS group, the levels of T-AOC, CO and GSH, the activities of CAT, GSH-Px and SOD as well as protein expressions of SOD1 and SOD2 were significantly decreased (*P* < 0.05, *P* < 0.01; **Figures 6A1–A6,B1,B2**), but the contents of ⋅OH, MDA, and GSSG were obviously increased (*P* < 0.05; **Figures 6C1–C3**) in the myocardial tissues of rats in SD + HS + HA group.

## Discussion

Myocardial hypertrophy is a cardiac compensatory change responding to the outside stimulus, demonstrated by increased myocardial cells volume, myocardial interstitial cell proliferation, increased extracellular matrix and other pathological changes. Long-term myocardial hypertrophy can lead to an increased myocardial oxygen consumption and reduce compliance of the heart eventually leading to heart failure or sudden death. Previous studies have shown that high-salt diet can lead to myocardial hypertrophy independent of blood pressure, thereby increasing the incidence of cardiovascular events (Mak et al., 2013; Katayama et al., 2014). However, the mechanism underlying high-salt-induced myocardial hypertrophy has not been clear yet.

Many studies showed that H_2_S had an important protective effect on a variety of cardiovascular diseases (Qipshidze et al., 2012; Nicholson et al., 2013; Polhemus et al., 2013; Pan et al., 2014). H_2_S could reduce myocardial remodeling caused by myocardial ischemia by increasing the activity of natriuretic peptide and improve myocardial remodeling by inhibiting myocardial fibroblast cell migration to myocardial fibroblasts (Lilyanna et al., 2015; Zhang et al., 2015). In smoking rat model and heart failure rat model, H_2_S also reduced myocardial remodeling and improved cardiac function (Mishra et al., 2010; Zhou et al., 2015). Our previous study showed that H_2_S could alleviate high salt-induced aortic remodeling and renal injury (Huang et al., 2015, 2016), but the protective effect of H_2_S on high-salt-induced myocardial hypertrophy was unclear. In the present study, we found that high-salt diet significantly reduced H_2_S content and CBS mRNA and protein expressions of myocardial tissues with significant myocardial hypertrophy in Dahl rats. At the same time, we also found that the mRNA expression of CSE was also downregulated, while no change of protein expression of CSE was observed in Dahl rats with high-salt intake. The regulation on the post-trancriptional level or translational level might lead to the differences in the mRNA and protein expressions of CSE in Dahl rats with high-salt intake (Greenbaum et al., 2003). However, there was no significant change in the above indexes in myocardial tissues of SD rats fed with high salt. The results showed that the down-regulation of H_2_S/CBS pathway induced by high-salt diet was closely correlated with myocardial hypertrophy. After H_2_S treatment, myocardial hypertrophy was significantly reduced in Dahl + HS group. The results demonstrated that H_2_S significantly improved high-salt-induced myocardial hypertrophy. In contrast, myocardial hypertrophy became obvious in SD rats treated with HA, an inhibitor of CBS, as compared with SD + HS rats without HA treatment. Taken together, the results demonstrated that H_2_S could attenuate high salt-induced myocardial hypertrophy.

However, protective mechanism by which H_2_S attenuated high salt-induced myocardial hypertrophy was unclear. Oxidative stress is a kind of pathological state, which refers to excessive oxygen free radicals and (or) intracellular antioxidant defense system damage, leading to excessive accumulation of oxygen free radicals and related metabolites, resulting in a variety of toxic effects on cells. Reactive oxygen species (ROS) include superoxide anion, ⋅OH and hydrogen peroxide (H_2_O_2_). Reactive nitrogen species (RNS) include nitric oxide (NO), nitrogen dioxide (NO_2_), and peroxynitrite (ONOO^-^). The antioxidant system in the body includes the following enzymes, such as SOD, CAT, GSH-Px and a variety of vitamins. Studies have shown that oxidative stress plays an important role in the formation of myocardial hypertrophy. Enhanced NADH/NADPH oxidase activity and increased production of ROS could lead to myocardial hypertrophy, and also the application of antioxidants such as GSH-Px could significantly reduce myocardial hypertrophy (Amin et al., 2001; MacCarthy et al., 2001; Shiomi et al., 2004). Studies showed that oxidative stress was involved in myocardial hypertrophy induced by high salt (Yu et al., 1998; Gao et al., 2011; Wu et al., 2016). While, H_2_S was reported to be an important anti-oxidant and play anti-oxidative role in the nervous system, cardiovascular system, respiratory system, and renal tissues, etc (Geng et al., 2004a; Yu et al., 2015; Huang et al., 2016; Zhang et al., 2016). However, whether H_2_S inhibited oxidative stress injury in the high-salt-stimulated myocardial hypertrophy was not fully understood.

Therefore, we detected oxidative stress and antioxidant stress indices in the myocardial tissues to see if the inhibitory effect of H_2_S on myocardial hypertrophy was related to the increase of anti-oxidative capacity. We found that high-salt significantly induced excessive oxidative stress in myocardial tissues of Dahl rats as demonstrated by the decreased levels of T-AOC, CO and GSH, the activities of antioxidant enzyme and protein expressions of SOD1 and SOD2, and elevated levels of ⋅OH, MDA, and GSSG. The above results indicated that high-salt induced oxidative stress injury of myocardial tissues in Dahl rats. The results of Dahl rats treated with NaHS showed that levels of T-AOC, CO and GSH, the activities of CAT, GSH-Px and SOD, and protein expressions of SOD1 and SOD2 in the myocardial tissues were increased with decreased the contents of ⋅OH, MDA, and GSSG. While HA upregulated all detected oxidative stress indices but downregulated all tested anti-oxidant stress indices in the myocardial tissues of SD rats with high-salt diet, mimicking the effect of high salt on oxidative stress injury in the myocardial tissues of Dahl rats. Therefore, the study demonstrated that H_2_S attenuated high-salt-induced myocardial hypertrophy in association with inhibiting oxidative stress of myocardial tissues in salt-sensitive rats.

The present study also had limitations. We demonstrated the effect of H_2_S on high salt-induced myocardial hypertrophy by using NaHS, an H_2_S donor, but we did not use different well known H_2_S donors and different experimental models to illuminate the beneficial effects of H_2_S on high salt-induced myocardial damage. It would be necessary and important to further explore the H_2_S-based treatment for high salt-induced vascular and myocardial damage in experimental and even clinical studies.

## Conclusion

In summary, the study demonstrated that H_2_S could attenuate high-salt-induced myocardial hypertrophy in Dahl rats. The protective mechanism might be related to the enhancement of antioxidant capacity and the inhibition of oxidative stress of myocardial tissues.

## Author Contributions

PH, CT, HJ and JD conceived and designed the experiment. PH, ZS, and WY performed the experiment, PH analyzed data and wrote the paper. YH, HJ, CT, and JD revised the manuscript. All authors have contributed to the final version and approved the publication of the final manuscript.

## Conflict of Interest Statement

The authors declare that the research was conducted in the absence of any commercial or financial relationships that could be construed as a potential conflict of interest.
